# Mysterious Bilateral Foot Pain in a Child With Crouzon Syndrome

**DOI:** 10.7759/cureus.36999

**Published:** 2023-04-01

**Authors:** Kyle Coombes, Madeleine Yeakle, Magda Kwiatkowska, Marcin Kwiatkowski

**Affiliations:** 1 School of Medicine, American University of the Caribbean, Cupecoy, SXM; 2 Pediatric Orthopedics and Traumatology, Gruca Teaching Hospital, Warsaw, POL; 3 Radiology, School of Medicine, American University of the Caribbean, Cupecoy, SXM

**Keywords:** craniosynostosis, orthopedics, genetics, pediatrics, crouzon syndrome

## Abstract

Crouzon syndrome (CS) is a rare autosomal dominant disorder that requires care from a multidisciplinary team and early surgical management to minimize complications. Despite the shared similarities across craniosynostoses, CS can be differentiated by the presence of normal bone development of the hands and feet and hypertelorism (large distance between the eyes). Other common features include midface hypoplasia, shallow orbits, ocular proptosis, and dental abnormalities including possible bifid uvula or V-shaped maxillary arch. In this report, we present a case of prolonged foot pain in a four-year and two-month-old boy with CS; we also engage in a brief review of the literature. The patient's physical exam and laboratory work were unremarkable on the initial presentation. Radiographic films showed signs of potential demineralization of bone tissue. He was prescribed calcium and vitamin D supplementation with complete resolution of his symptoms at the three-month follow-up visit.

## Introduction

Crouzon syndrome (CS; craniofacial dysostosis type I, MIM #123500) is a rare genetic disorder, occurring in approximately 1/25,000 births worldwide. It commonly presents with premature fusion of the coronal and sagittal sutures [1,2]. Other common features include midface hypoplasia, hypertelorism (large distance between the eyes), shallow orbits and ocular proptosis, and dental abnormalities, including possible bifid uvula or V-shaped maxillary arch [1,2].

CS is the result of an autosomal dominant disorder at the chromosomal locus, 10q25.3-q26, of the FGFR2 gene, with more than 30 possible mutations documented to date [2]. Craniosynostotic syndromes result from mesenchymal and ectodermal abnormalities, the latter playing a critical role in brain embryogenesis [3]. Premature fusion of cranial sutures limits brain growth perpendicular to that suture, forcing the brain to grow in the direction of any open sutures [4]. The result is the distinctive distortion of the cranial vault and skull-base architecture seen in most craniosynostoses. Differential diagnoses for CS include simple craniosynostosis, Apert syndrome, Carpenter syndrome, Saethre-Chotzen syndrome, and Pfeiffer syndrome [2].

While many craniosynostoses present with skull-base abnormalities and midface hypoplasia, CS is differentiated by a lack of hand or foot abnormalities and hypertelorism [3]. The neurological manifestations of CS may range from normal intelligence to moderate intellectual disability, secondary to the degree of intracranial volume reduction [2,3]. Early radiologic evaluation is essential not only for identifying osseous abnormalities but also to assess for ectodermal malformations such as Dandy-Walker syndrome or schizencephaly [3]. Additionally, patients with CS are also at increased risk for elevated intracranial pressure, hearing defects, upper airway obstruction, and obstructive sleep apnea [3,5]. A thorough clinical evaluation with a multidisciplinary approach is critical to limit complications and improve patient outcomes [3,5,6].

## Case presentation

A four-year and two-month-old Caucasian boy with CS presented for the evaluation of his ongoing complaints of bilateral foot pain of a year's duration. History from the mother and patient revealed frequent night-time awakenings secondary to crying, which had eventually begun to affect his sleep. The patient had been born at 39 weeks vaginally to a G3P2, with Apgar scores of 10 and 10 at one and five minutes, respectively, and weighing 3200 grams. There had been no complications associated with the delivery. However, the pregnancy had been complicated by gestational diabetes, hypothyroidism, thrombocytopenia, and maternal urinary tract infection. At the age of six weeks, an ultrasound screening of the patient for developmental dysplasia of the hips had revealed Graf scores of IB/IIA in the right hip and IIB in the left hip. He had been placed in a Pavlik harness for six weeks.

By the age of three, he had undergone an occipital-suboccipital craniotomy with the implementation of KLS-Martin 30-mm distractors that were removed several months later. He had been diagnosed with myopia (-0.75), astigmatism, and bilateral strabismus early on in life. His bilateral nasal meatus had been critically narrowed leading to sleep apnea. He had decreased auditory acuity in the left ear and was also found to have a butterfly-type of C3 vertebrae on spinal MRI. There was no family history of CS or other genetic disorders.

His physical examination was largely unremarkable with a fully active and passive range of motion of the lower extremities. The feet were symmetrical with a typical mild-flexible flatfoot appearance. There was no swelling, erythema, or tenderness to palpation on examination of the lower extremities. Upon evaluation of the spine and on Adam’s test, there was a slight tilt of the pelvis, indicative of a possible lower limb length discrepancy. The lower limb measurements were performed in the supine position, with the joints in a neutral position and the pelvis squared. A tape measure was extended from the anterior superior iliac spine to the medial malleolus, and a 1-cm limb length discrepancy was appreciated on the right. The patient’s facial features were pathognomonic for CS. He was in the 80th percentile for height and weight. There were no neurological deficits appreciated at the time of his visit. 

**Figure 1:**
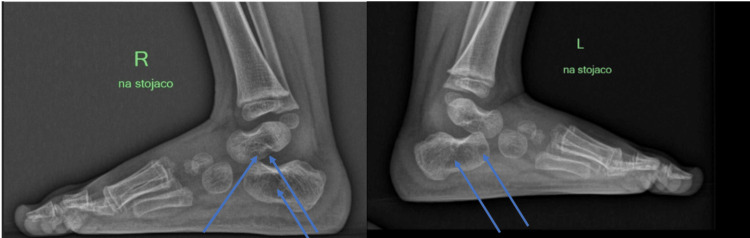
Lateral X-rays of the feet before treatment The arrows indicate poor bone density with bone cysts and cavitation

**Figure 2:**
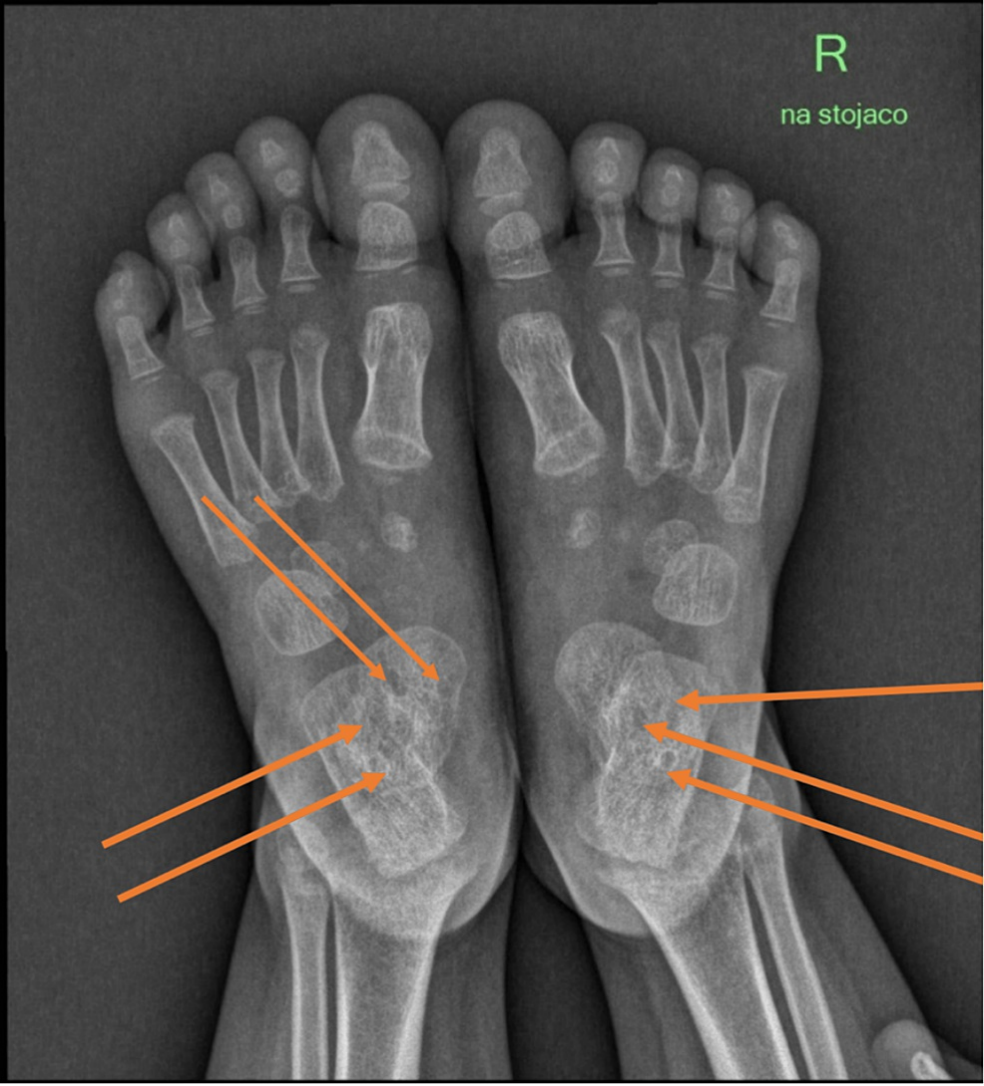
Anterior-posterior (AP) X-rays of the feet before treatment The arrows highlight the poor bone density areas with bone cysts and cavitations

**Figure 3:**
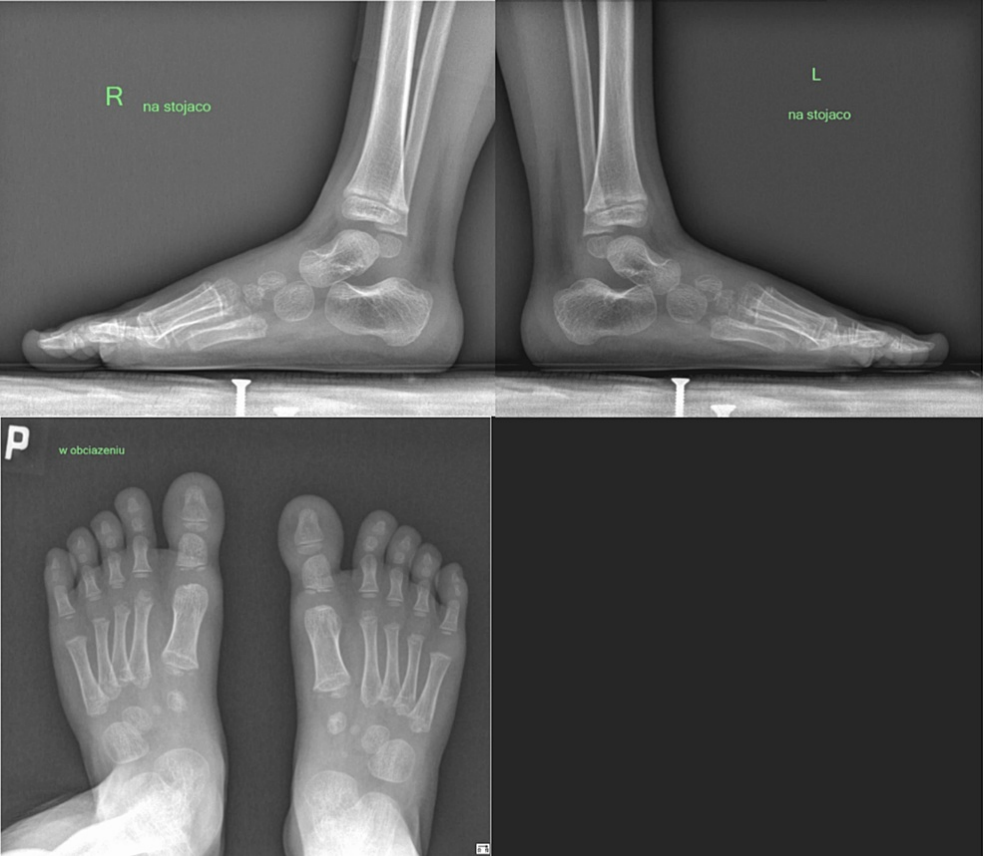
Lateral and standing anterior-posterior (AP) X-rays of the feet post-treatment showing normal bone structure and quality

**Figure 4:**
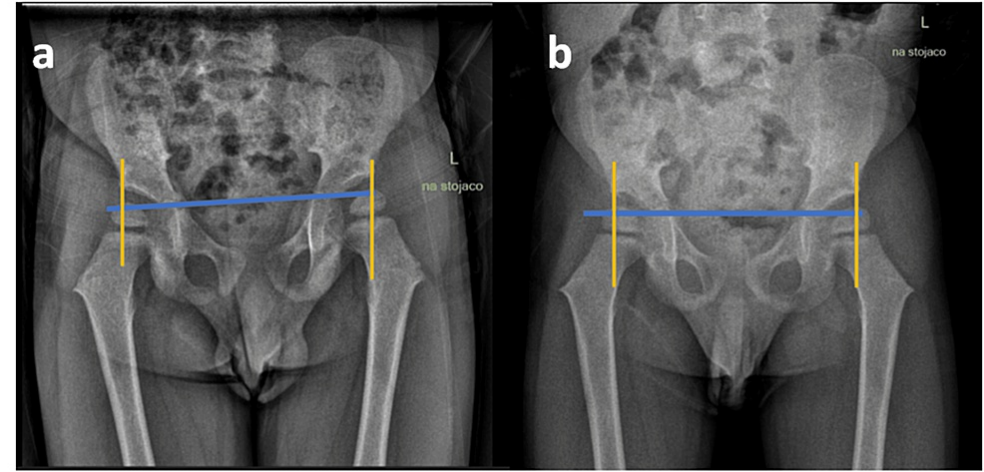
Anterior-posterior (AP) X-ray of the hips pre-treatment (a) and post-treatment (b) Hilgenreiner and Perkin's lines are drawn to evaluate the DDH. The Hilgenreiner line is drawn through the triradiate cartilages. The Perkin's line is drawn perpendicular to the Hilgenreiner line at the margin of the bony acetabulum. The discrepancy in the lower limbs' length is visible DDH: developmental dysplasia of the hip

Routine bloodwork, including calcium and vitamin D levels, was performed and found to be within normal limits. An ultrasound of the feet showed no abnormalities; however, radiographic imaging revealed decreased mineralization of the bones bilaterally with visible cysts (Figures 1-3). Additional X-rays of the pelvis were obtained, demonstrating decentration of the left hip joint (Figure 4a).

The patient was referred to the Department of Pediatric Osteoporosis and Metabolic Bone Disease for further evaluation and serial densitometry in six-month periods. The decision was made to defer densitometry in favor of starting a regimen of supplements including vitamin D3 1500 IU and calcium 500 mg daily with a follow-up in three months. On his return visit, his symptoms had completely resolved, and no limb length discrepancy or pelvic tilt was observed. Densitometry (DEXA) scan performed four months post-supplementation revealed a Z-score of -0.7. A repeat DEXA scan at six months showed a Z-score of -0.5. We were unable to obtain pre-treatment Z-scores for comparison due to his age at the time. Radiographic films performed at six months revealed normal bone structure with improved hip findings, and no decentration of the hip was noted (Figure 4). Repeat imaging was conducted at the one-year follow-up and confirmed the resolution of lower limb abnormalities.

## Discussion

CS, first described by the French neurologist Octave Crouzon in 1912, is the most common craniosynostosis syndrome and is usually diagnosed at birth based on notable craniofacial abnormalities and positive family history [7-9]. However, its diagnosis can be incidental if spontaneous mutations arise and warrant genetic testing [8]. FGFR2 is a transmembrane protein receptor responsible for osteoblast differentiation and can be found in perichondrial and periosteal tissue, trabecular bone, cartilage of the cranial base and growth plate, and osteoprogenitor cells of cranial sutures [8,10].

Radiographic films, which most commonly display peri-sutural sclerosis, reduced serration, and bony bridging and/or the absence of the suture altogether, are helpful in determining the degree of skull-base abnormalities and identifying sequelae of the syndrome [8]. Early surgical intervention during the first two years of life is necessary to ensure a good prognosis, improve life expectancy, and reduce the degree of intellectual disability [7,8].

Surgical management typically involves several phases targeting specific structures. The first phase usually involves remodeling the fronto-orbital area and releasing stenotic sutures so that proper brain growth can occur [7,11]. Once the child reaches late adolescence, corrections including midfacial, orthodontic, and orthognathic surgery are performed to ensure a more normal cosmetic appearance [7,11].

## Conclusions

While there is no cure for CS yet, early surgical management remains the mainstay of treatment and can prevent serious complications, including deafness, vision loss, intellectual disability, developmental delays, and increased intracranial pressure. In our case, while the patient's pain was not well described, it could be secondary to the cystic areas appreciated on radiographic analysis as these are part of the osteoporotic presentation. Hence, vitamin and mineral supplementation in these patients may provide symptomatic relief and be effective in improving bone quality over time.
